# Vasculitis and neurobrucellosis: Evaluation of nine cases using radiologic findings

**DOI:** 10.1002/brb3.947

**Published:** 2018-03-09

**Authors:** Sule Aydin Turkoglu, Siddika Halicioglu, Fatma Sirmatel, Murside Yildiz, Nebil Yildiz, Serpil Yildiz

**Affiliations:** ^1^ Department of Neurology Abant Izzet Baysal University Medical Faculty Bolu Turkey; ^2^ Department of Radiology Abant Izzet Baysal University Medical Faculty Bolu Turkey; ^3^ Department of Infectious Diseases Abant Izzet Baysal University Medical Faculty Bolu Turkey; ^4^ Department of Intensive Care Abant Izzet Baysal University Medical Faculty Bolu Turkey

**Keywords:** magnetic resonance imaging, neurobrucellosis, stroke, vasculitis

## Abstract

**Background:**

Brucellosis is an important multisystemic disease with many different clinical symptoms, and its early diagnosis and treatment are possible. Neurobrucellosis (NB) is a rare but serious finding of brucellosis. Brucella can be seen as meningitis and encephalopathy, and it can cause cranial nerve pathologies, vascular syndromes, myopathy, spinal diseases, and psychiatric disorders. In NB, vascular syndromes secondary to inflammation are rarely seen. Here, we present nine young patients with vascular and nonspecific neuropsychiatric findings who had NB as the etiology of stroke.

**Methods:**

Nine patients who were admitted to our Neurology Clinic between 2012 and 2017 for various reasons in whom brucellosis was found in the etiology were retrospectively studied. The patients' symptoms, physical examination, laboratory and radiographic findings, treatments, and treatment responses are discussed.

**Results:**

Of the nine patients who presented to our clinic in the 4‐year period, five were female. The average age was 49 years. Five patients had small vessel vasculitis, three had great vessel vasculitis, and one had meningoencephalitis and pons abscess. Two patients had granuloma, and one had an aneurysm.

**Conclusions:**

We aimed to present our cases due to the fact that this disease should be kept in mind in the differential diagnosis of patients with stroke and similar neuropsychiatric findings.

## 1 INTRODUCTION

Brucellosis, caused by gram‐negative bacteria of the genus Brucella, is a zoonotic disease transmitted through the consumption of undercooked meat, milk, and dairy products of infected animals, such as sheep, goats, cattle, buffalo, and pigs, and transmitted to people who have close contact with the secretions of infected animals. There are no distinctive features specific to Brucella infections. It is often a diagnostic complication because it is a multisystemic disease that occurs in different clinical forms. Brucellosis is an infectious disease with a high rate of morbidity, which can become chronic, and affects thousands of people every year and causes loss of workforce potential (Ay, Tur, & Kutlay, 2010). The incidence of central nervous system (CNS) involvement is 0.5%–25%. Neurobrucellosis (NB) can occur with acute or chronic meningitis, meningoencephalitis, myelitis, radiculitis, cranial nerve involvement, spinal or brain abscess, subarachnoid hemorrhage, ischemic or hemorrhagic stroke, and neuropsychiatric symptoms. Optic, oculomotor, abducens, facial, and vestibulocochlear nerves are the frequently involved cranial nerves. These tend to involve the vestibulocochlear nerve, leading to sensorineural hearing loss. CNS involvement is due to inflammation caused by both a direct bacterial effect and the effect of cytokines and endotoxins on the peripheral nerves, spinal cord, meninges, brain, or vascular structures (Akdeniz, Irmak, Anlar, & Demiröz, 1998; Ay et al., 2010; Bucher, Gaustad, & Pape, 1990; Sanchez‐Sousa et al., 1990; Young, 2006; Zheng et al., 2018). Diagnosis in NB is based on the production growth of a bacterium in the cerebrospinal fluid sample (CSF) or the presence of any titrated Brucella antibodies in CSF and the presence of abnormal CSF (more than 10 cell counts, decrease in CSF glucose, increase in CSF protein) (Bucher et al., 1990; Guven et al., 2013; Sanchez‐Sousa et al., 1990). NB has a good prognosis with early diagnosis and treatment. Mortality in NB has decreased to 0%–5.5% after appropriate antibiotherapy in recent years, but persistent deficits such as hearing loss are more frequent (Mousa et al., 1986). Brucellosis is still a major public health problem in developing countries such as our country. NB will be discussed in conjunction with the presentation nine patients who had clinical symptoms of NB. In our country, we have extensive livestock and prevalent infectious diseases. We aimed to emphasize that NB should be kept in mind in the differential diagnosis of many diseases that involve cerebrovascular diseases, demyelinating diseases, polyneuropathy, radiculopathy, motor neuron disease, and the CNS.

## 2 METHOD

Nine patients who were admitted to our Neurology Clinic between 2012 and 2017 for various reasons in whom brucellosis was found in etiology were retrospectively studied. The patients' symptoms, physical examination, laboratory and radiographic findings, treatments, and treatment responses are discussed.

Diagnosis of NB: (1) compliance of the patient's clinical findings with neurobrucellosis; (2) detection of typical CSF findings in laboratory tests; (3) positive serologic tests of the Brucella standard agglutination test in blood and/or CSF, positive Coombs test of ≥1/80 titer in blood and/or CSF; (4) detection of Brucella in blood and/or CSF culture, and the absence of a more suitable alternative diagnosis.

### 2.1 Case 1

**Figure 1:**
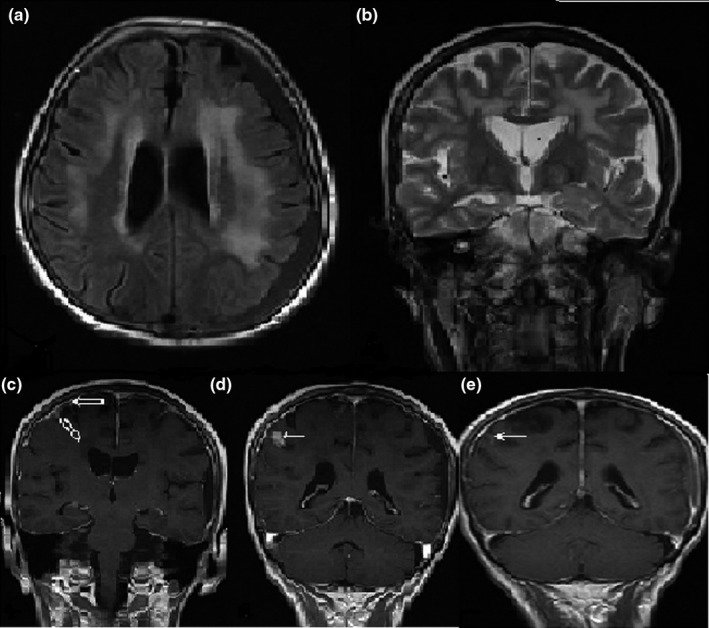
Axial FLAIR (a) and coronal T2‐weighted (b) images of patient with sensorineural hearing loss showed diffuse hyperintense signal changes bilaterally in the subcortical and periventricular deep white matter and the subdural hygroma on the left. In postcontrast coronal (c) series, diffuse dural and leptomeningeal (open arrow) enhancement and right frontal granuloma formation (white arrow) (d) were observed; After treatment, the granuloma size decreased (e) but leptomeningeal enhancement continued

A 56‐year‐old woman who worked in animal husbandry reported symptoms of headaches, night sweats, joint aches, speech impairment, personality change, forgetfulness, depression, and hearing loss, which she had had for the last 4 years. At presentation, the patient had newly developed loss of muscle strength in the left arm and leg. In a hearing test performed 2 years ago, the presence of moderate sensorineural hearing loss in the left ear and total hearing loss in right ear was detected. Diffuse T2W (T2‐weighted) hyperintense signal changes were detected in cranial images of the subdural hygroma and right frontal hygroma using leptomeningeal enhancement and postcontrast images (Figure 1a–d). In the study for etiology, Brucella Rose Bengal (+) and Brucella agglutination: 1/160 were detected in the blood. With the exception of Brucella Rose Bengal (+) and Brucella agglutination (1/160) in the CSF study, no finding was detected. Acetylsalicylic acid (ASA)–clopidogrel combination therapy and antibiotic therapy improved the patient's clinical condition completely. Cranial magnetic resonance imaging (MRI) showed partial regression (picture 1e). The patient was evaluated as having neurobrucellosis small vessel vasculitis.

### 2.2 Case 2

**Figure 2:**
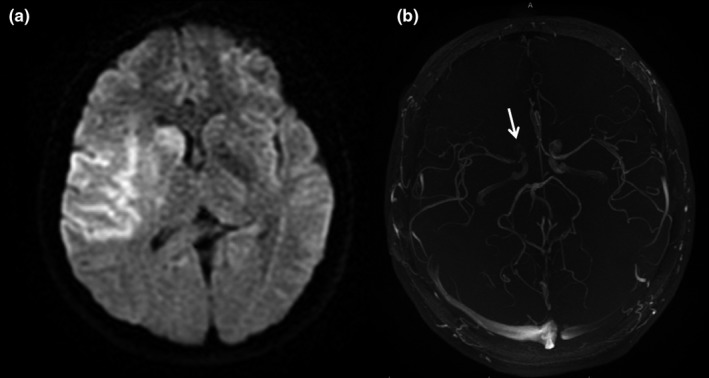
In a patient with stroke, in diffusion‐weighted images (a), hyperintensity consistent with acute infarction was observed in the right MCA territory. A 3D TOF MR angiography showed (b) irregularity (arrow) in the right ICA supraclinoid segment and loss of signal in the first segment of the right midcerebral artery

A woman aged 33 years. Acute infarction in the right middle cerebral artery (MCA) was observed in cranial computed tomography (CCT), which was performed because of headache, joint pain and nausea, vomiting, and loss of power in the left arm and leg. On detection of acute infarction in the right MCA, three‐dimensional time‐of‐flight (3D TOF) magnetic resonance (MR) angiography was taken and signal loss was observed in the right internal carotid artery (ICA) and right MCA (Figure 2). In the examination for the etiology of the stroke of the young patient, there were no external features except Brucella Rose Bengal (+) and Brucella agglutination (1/160). Cerebral edema developed in the follow‐up CT of the patient who lost consciousness and was disturbed in general condition. Despite receiving treatment for the cerebral edema, the patient died on the 5th day of admission. The patient's neurobrucellosis was evaluated as great vessel vasculitis.

### 2.3 Case 3

**Figure 3:**
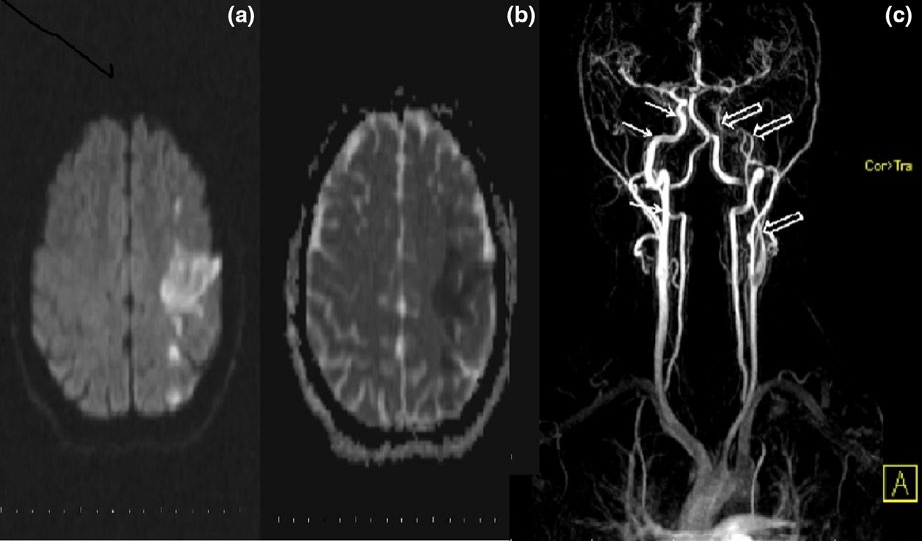
In patient with dysarthric speech and right hemiparesis, left frontoparietal hyperintense acute infarct areas in the diffusion‐weighted image (a) and (b) restriction in the ADC map were observed. MR angiography (c) showed that the left ICA (open arrows) calibration was significantly reduced, and there was no contrast filling in the supraclinoid segment

A 37‐year‐old man presented with headache, dizziness, speech impairment, and loss of strength in the right half of his face, right arm, and leg. The patient had dysarthric speech, loss of the right nasolabial fold, drooping of the right eyelid, and facial asymmetry. The right upper and lower extremity muscle strength were 1/5 and 3/5, respectively. Cranial MR revealed a lesion compatible with an acute infarct and limited left frontoparietal diffusion (Figure 3a,b). In the study for etiology, there were no extra features except positivity for Brucella Rose Bengal and Brucella agglutination: 1/320. Antibiotic therapy was initiated. Contrast‐enhanced MR angiography of the neck and brain revealed mild‐grade stenosis at the exit of the left main carotid artery, a contrast signal surrounding the exit of the left main carotid artery, and a contrast signal surrounding the exit of the brachiocephalic artery, suggesting that this nonspecific appearance may be due to vasculitis (Figure 3c). On the right side, there were no traits other than frust hemiparesis. The patient was evaluated for large vessel vasculitis of neurobrucellosis.

### 2.4 Case 4

**Figure 4:**
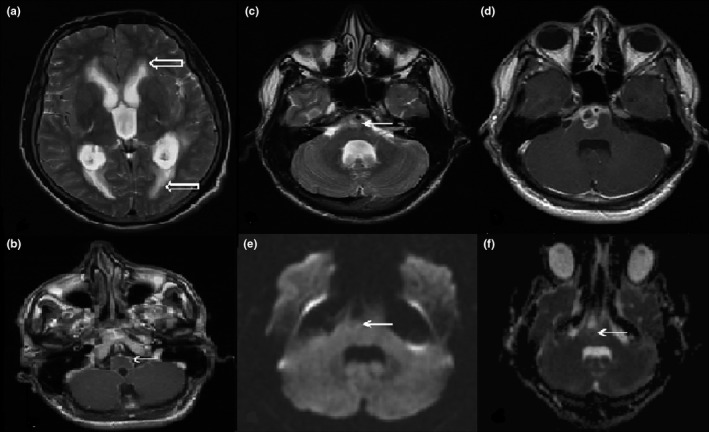
In patient with meningoencephalitis, first MRI axial T2‐weighted and postcontrast axial T1‐weighted images demonstrated (a) a tetraventricular hydrocephalus, transependymal CSF transition, and (b) leptomeningeal enhancement especially in the basal sections and brainstem levels. Follow‐up (12th day) T2‐weighted MRI revealed heterogeneous hyperintense signal changes in the pons and right hemisphere (c). In the repeated MRI after worsening of the patient's clinical findings, (24th day) there occurred a heterogenous area with lobulated peripheral contrast enhancement in the T1‐weighted scans (d) with minimal hyperintensity and restriction in the diffusion and ADC map sequences (e–f) in the pons

A man aged 25 years presented with symptoms of nausea, vomiting, dizziness, confusion, and meaningless speech, in addition to weakness, fatigue, loss of appetite for the last 4–5 months. In the patient, who had a tendency to sleep, neck stiffness and bilateral papillary edema were detected and lumbar puncture (LP) was performed for meningoencephalitis as the preliminary diagnosis. LP revealed high CSF protein, 30 cells, and negative culture. Cranial MRI revealed triventricular hydrocephalus and leptomeningeal contrast enhancement (Figure 4a,b). Brucella Rose Bengal (+) and Brucella agglutination: 1/160 were detected in routine blood tests performed for the etiology. The patient was treated successfully with antibiotic therapy and was discharged to complete the treatment for 6 months. On the 12th day and on the 24th day of the onset of treatment, the patient was admitted with worsening of the clinical findings and loss of strength in the left arm and leg because the patient had stopped the treatment. A neurologic examination revealed left central facial paralysis, left upper and lower muscle strength was 4/5, and the left plantar response was extensor. In the cranial MR images of the patient, a lesion compatible with abscess was detected in the right half of the pons (picture 4c–f). The antibiotic therapy was rescheduled, and complete improvement was seen in the clinical condition of the patient. The patient was evaluated as having meningoencephalitis and pons abscess of neurobrucellosis.

### 2.5 Case 5

A 53‐year‐old man presented with electrical sensation, tingling, headache, fatigue, and fever on the face and the entire body; these signs were more prominent on both upper limbs. The patient, who had no clinical findings in the neurologic examination on referral, was found to have had hearing loss in the right ear and widespread joint pain for the past 2 years. On cranial MRI imaging, T2W hyperintense ischemic gliotic lesions with a diffuse nodular appearance were detected. In the study for etiology, Brucella Rose Bengal was (+), and Brucella agglutination was detected as 1/160. The patient refused to undergo LP. Following treatment with antibiotic therapy, the patient's symptoms were fully improved. The patient's neurobrucellosis was evaluated as small vessel vasculitis.

### 2.6 Case 6

**Figure 5:**
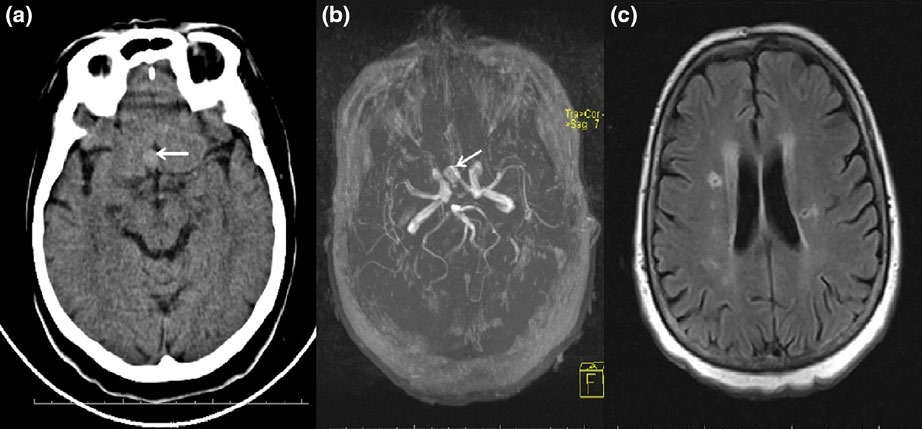
In case 6, axial cranial CT without contrast revealed a nodular hyperdense (arrow) suspicious appearance in terms of aneurysm in the anterior communicating artery location (a); the aneurysm was confirmed with 3D TOF MR angiography (b). Bilateral periventricular T2‐weighted hyperintense (c) signal changes in the deep white matter were also observed

A 65‐year‐old woman developed speech impairment while taking ASA 100 mg due to coronary artery disease. The patient described having fatigue and drowsiness for the last few months. A cranial diffusion MRI study showed acute limitation of the diffusion in the right precentral gyrus, cranial MRI revealed lesions compatible with small vascular disease, and MR angiography revealed a saccular aneurysm in the anterior communicating artery (Figure 5). For the etiopathogenesis of the cardiac embolism, echocardiographic and cardiologic examinations revealed no positive findings. Carotid and vertebral artery Doppler ultrasonography (USG) were within normal limits in terms of thromboemboli. Brucella Rose Bengal (+) and Brucella agglutination: 1/80 were detected in the blood tests performed. The patient was treated with antibiotics and was evaluated as having great vessel vasculitis of neurobrucellosis.

### 2.7 Case 7

**Figure 6:**
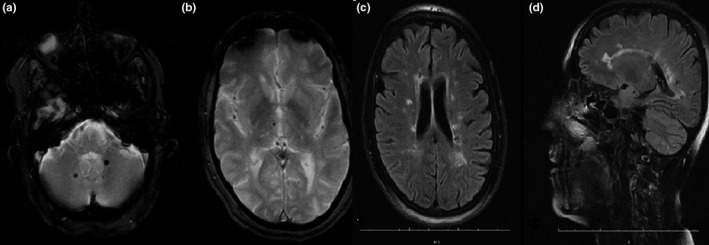
In case 7, gradient echo MRI showed signal loss consistent with multiple hemosiderin deposits (hypertensive microhemorrhage? amyloid angiopathy?) in both cerebellar hemispheres (a), thalamus (b), pons, right hemisphere, left lentiform nucleus, and left frontal gray–white matter. In axial (c) and sagittal (d) FLAIR sequences, hyperintense signal changes, some of which were located vertically to the callososeptal interface, were observed in bilateral periventricular deep white matter

A 50‐year‐old woman was found to have received inadequate treatment for Brucella 7 years ago. The cranial MR of the patient, who had progressive deterioration of walking and difficulty in walking without support for 2 years, was found to show an appearance compatible with a large number of demyelinating plaques and amyloid angiopathy (Figure 6). In the tests for etiology, there was no extra characteristic detected except Brucella Rose Bengal (+) and Brucella agglutination: 1/160. CSF was normal. Brucella treatment was initiated. In the follow‐up, a partial improvement was observed in his symptoms. The patient's neurobrucellosis was evaluated as small vessel vasculitis.

### 2.8 Case 8

**Figure 7:**
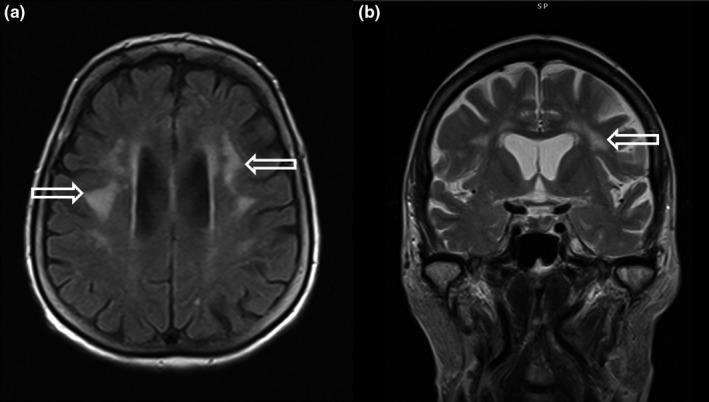
In case, 8, axial (a) and coronal (b) T2‐weighted images showed nodular–patchy T2‐weighted hyperintense signal changes (open arrows) in bilateral periventricular deep white matter

A 77‐year‐old woman was referred with gait disturbance, sweating, and widespread body pain. There were no findings except Brucella Rose Bengal (+) blood and Brucella agglutination: 1/160. In addition to findings consistent with common vascular disease, there were significant T2W hyperintense lesions in the bilateral frontal lobes in cranial MR, which were significant for neurobrucellosis (Figure 7). In the audiometric examination of the patient, a slight sensorineural hearing loss on the right side and a mixed‐type hearing loss on the left side were detected. In the CSF examination, there was no positive finding. The treatment of the patient is still ongoing. The patient's neurobrucellosis was evaluated as small vessel vasculitis.

### 2.9 Case 9

**Figure 8:**
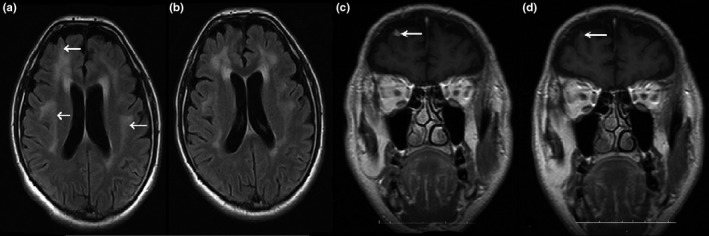
In case 9, axial T2‐weighted images demonstrated hyperintense signal change areas in (a) the bilateral subcortical and periventricular deep white matter. Postcontrast coronal T1‐weighted (c) images showed a white arrow‐marked granuloma‐like appearance with significant extra‐axial enhancement near the right frontal lobe. In the follow‐up images taken 2 months after the onset of treatment, volume and signal regression (b) and decrease in the size of the (d) granuloma (white arrow) were observed

A man aged 44 years who underwent cranial MR investigations due to extensive muscle and joint pain in 2013, fever and weakness in 2014, diffuse body pain and hearing loss in 2014, headache and epileptic seizures in 2016, extensive T2W hyperintense lesions and right frontal contrast enhancement and a granuloma‐compatible lesion, which had partially regressed with treatment, were detected (Figure 8). The audiometric examination performed was compatible with bilateral moderate hearing loss of mixed type. In the studies for etiology, no findings except Brucella Rose Bengal (+) blood and Brucella agglutination (1/2560) were detected. In the CSF examination, Brucella tube agglutination was 1/20, and a partial regression of the cranial MR findings was observed after the patient received antibiotic treatment (Figure 8). The patient's neurobrucellosis was evaluated as small vessel vasculitis and granuloma.

## 3 RESULTS

Of the nine patients who applied to our clinic over the 4‐year period, five were female. The average age was 49 years (56–33–37–25–53–65–50–77–44 years). Two patients worked with livestock, and one patient had a history of consuming cheese made from uncooked milk. Two patients had a history of inadequate Brucella treatment.

Although the primary symptoms of the patients on admission were stroke, unconsciousness, gait disturbance, head and joint pain, fatigue, depression, and hearing loss, we additionally detected fever in five cases, hemiparesis in four cases, confusion in two cases, hearing loss in four cases, dysarthria in one case, and progressive gait disturbance in one case in the physical examination. One patient had nuchal stiffness, hepatosplenomegaly, and lymphadenopathy. In one patient, there was a widespread electrical feeling throughout their body. There was no characteristic feature in the tests performed for the etiology of cardiac embolism, thromboembolism, and other cause of vasculitis. There were no positive findings regarding blood pressure, heart rate, echocardiography, carotid and vertebral artery Doppler USG examinations, and electrocardiography (ECG) and posteroanterior (PA) chest radiographs. The laboratory blood values of the patients are shown in Table 1, CSF findings in Table 2, and cranial imaging findings in Table 3. Five of these patients had small vessel vasculitis, three had great vessel vasculitis, and one had meningoencephalitis and a pons abscess. Two of these patients had a granuloma, and one had an aneurysm.

**Table 1:** Laboratory findings of patients

Serum	WBC	CRP	ESR	LDH	GGT	AST	ALT	RB serum	BA serum
Case 1	6,800	2.1	34	408	118	104	38	(+)	1/160
Case 2	10,200	0.2	15	134	19	14	10	(+)	1/320
Case 3	10,000	2.2	16	181	47	20	22	(+)	1/320
Case 4	2,261	79.3	14	287	20	14	10	(+)	1/160
Case 5	6,667	0.1	1	214	10	20	15	(+)	1/160
Case 6	7,380	10.9	30	210	14	18	20	(+)	1/80
Case 7	6,840	1.4	1,4	218	29	18	13	(+)	1/160
Case 8	7,018	3	3	168	14	16	12	(+)	1/80
Case 9	6,200	0.5	13	212	15	19	19	(+)	1/2560

ALT, alanine aminotransferase U/L; AST, aspartate aminotransferase U/L; CRP, C‐reactive protein mg/dl; ESR, erythrocyte sedimentation rate mm/saat; GGT, gamma glutamyl transferase U/L; LDH, lactate dehydrogenase U/L; WBC, white blood cell/mm^3^.

**Table 2:** CSF findings of patients

Case	CSF/serum glucose, mg/dl	CSF/serum glucose oran	CSF/serum protein, mg/dl	CSF/serum albumin, ug/ml	CSF/serum, LDHU/L	CSF/serum chloride, mmol/L	CSF rose Bengal	CSF Brucella agglutination	Culture blood and CSF
1.	30/73	0.41	272/6400	160/3400	34/383	110/97	(+)	1/160	(−)
2.	—	—	—	—	—	—	—	—	(−)
3.	58/139	0.42	87/7500	>500/4000	33/180	130/102	(−)	(−)	(−)
4.	57/108	0.53	618/6500	>500/4700	108/287	102/96	(−)	(−)	(−)
5.	—	—	—	—	—	—	—	—	(−)
6.	—	—	—	—	—	—	—	—	(−)
7.	63/113	0.56	15.9/6700	9,5/3900	11/194	127/105	(−)	(−)	(−)
8.	78/124	0.63	30/6400	—	19/168	124/102	—	—	(−)
9.	14/84	0.17	243/6600	176/3900	65/261	119/109	(+)	1/1280	(−)

BA, Brucella agglutination test; CSF, cerebrospinal fluid; LDH, lactate dehydrogenase; RB, Rose Bengal test.

(+): Positive; (−): Negative; (—): Cases with no CSF sample.

**Table 3:** Cranial imaging findings of patients

Case	Cranial imaging findings	Diagnosis
1	MRI: common T2W hyperintense signal change, subdural hygroma, and right frontal hygroma on postcontrast images with leptomeningeal contrast enhancement (Figure 1).	Neurobrucellosis small vessel vasculitis and granuloma
2	3D TOF MR angiography showed signal loss in the right ICA and right MCA (Figure 2).	Neurobrucellosis great vessel vasculitis
3	Lesion compatible with acute infarct that shows diffusion limitation in left frontoparietal region on MR. MR angiography showed a mild stenosis at the exit of the left main carotid artery, a contrast signal surrounding the exit of the left main carotid artery, and surrounding the brachiocephalic artery outlet (Figure 3).	Neurobrucellosis great vessel vasculitis
4	On cranial MRI, triventricular hydrocephalus and leptomeningeal contrast enhancement were detected, and a lesion consistent with abscess was detected in the right half of the pons (Figure 4).	Neurobrucellosis meningoencephalitis and pons abscess
5	Cranial MRI imaging revealed T2W hyperintense ischemic gliotic lesions of diffuse nodular appearance	Neurobrucellosis small vessel vasculitis
6	Cranial diffusion MRI revealed acute restriction of diffusion in the right precentral gyrus, cranial MRI revealed lesions compatible with small vessel disease, and saccular aneurysm was detected in the anterior communicating artery on MR angiography (Figure 5).	Neurobrucellosis great vessel vasculitis and saccular aneurysm
7	Widespread and large numbers of demyelinating plaques and amyloid angiopathy on Cranial MR (Figure 6).	Neurobrucellosis small vessel vasculitis
8	Cranial MR reveals T2W hyperintense lesions in bilateral frontal lobes in addition to findings consistent with diffuse small vascular disease and is significant for neurobrucellosis (Figure 7).	Neurobrucellosis small vessel vasculitis
9	Cranial MR reveals widespread T2W hyperintense lesions and a granuloma‐compatible lesion with right frontal contrast involvement, and it partially regresses with treatment (Figure 8).	Neurobrucellosis small vessel vasculitis and granuloma

3D TOF MR angiography, three‐dimensional time‐of‐flight magnetic resonance angiography; MRI, magnetic resonance imaging; T2W, T2‐weighted.

Although the serum Brucella agglutination test was positive, blood and CSF cultures were negative in all cases. CSF samples were not obtained in three patients; one patient died in the acute phase, and two patients did not allow samples to be obtained. The CSF cell count of the first patient was performed, and 100 cells/mm were detected. The leukocyte types in the CSF were 70% neutrophils and 16% lymphocytes. The cell count of the fourth case was performed, 30 cells/mm were detected, and lymphocytes were predominant. In the 10th case, 34 cells were detected per millimeter cubed. Polymorphonucleocytes (PMLs) were predominant. In the other cases, there were no cells in the CSF.

Doxycycline treatment was replaced with ciprofloxacin due to gastrointestinal tract (GIT) symptoms in the first case. Streptomycin was started in the third case because of GIT symptoms due to rifampicin treatment. Our second patient, who came with signs of acute cerebrovascular disease, died before the serology results and antibiotherapy. The fourth patient developed an abscess in the pons due to irregular treatment after meningoencephalitis. There was regression in the symptoms of fifth case in the follow‐up. The seventh case had previously undergone inadequate treatment and had disease progression. No treatment was initiated in the sixth case because they had low serum titers of serum Brucella agglutination levels; this patient was recommended to be followed up with physical examination and titration findings. Long‐term triple therapy was planned for the seventh patient who had previously been treated irregularly. In the other patients, triple antibiotic therapy was initiated and they are still under monitoring.

## 4 DISCUSSION

Brucellosis is a zoonotic infectious disease that is still seen, especially in endemic areas. In endemic countries, the transmission route is often unpasteurized infected milk consumption, whereas it is dependent on occupational exposure in developing countries. Occupations in the high‐risk group are livestock farmers, butchers, and veterinary and laboratory workers (Ay et al., 2010). The frequency of NB with CNS involvement is approximately 5%–7% of all cases of brucellosis (Buzgan et al., 2010; Karsen, Koruk, Duygu, Yapici, & Kati, 2012). The age of detection of brucellosis in endemic areas varies from 13 to 40 years (Ay et al., 2010; Aydın Teke et al., 2015; Buzgan et al., 2010; Gul, Erdem, & Bek, 2009; Haji‐Abdolbagi, Rasoulinezhad, Jafari, Hasibi, & Soudbakhsh, 2008). In our study, in which nine patients with NB were examined, the mean age of the patients was 49 years. The average age in our study was high compared with the literature. This may be due to the fact that some of the cases were chronic and had undergone inadequate therapy or the diagnosis of patients had been delayed due to nonspecific symptoms. This may have led to the presentation of these patients with stroke because of the delay in diagnosis. NB should be kept in mind, especially in cases of nonspecific and neuropsychiatric symptoms at a young age. Early diagnosis may prevent the development of permanent damage in these patients.

In a study of 1028 cases of brucellosis, the most frequent symptoms were arthralgia (73.7%), fever (72.2%), fatigue (71.2%), sweating (64.8%), weight loss (42.4%), myalgia (36.1%), and tremor (33.9%) (Buzgan et al., 2010). In the study of Gül et al., the most common symptoms of NB were headache (57%), fever (57%), sweating (30%), weight loss (28%), and back pain (23%); weakness (15%) and hearing loss (10%) were found to be rare symptoms (Gul et al., 2009). Headache, fever, tremor, fatigue, and unconsciousness were found to be frequent in the study of Abdolbagi et al., and they were reported at rates of 55%, 49%, 29%, and 26%, respectively, whereas paraplegia, visual disturbances, ataxia, and seizures were rare findings (Haji‐Abdolbagi et al., 2008). In our study, three of the nine patients presented with stroke and were evaluated as having great vessel vasculitis. Five patients with symptoms of headache, muscle and joint pain, fatigue, and depression had nonspecific ischemic gliotic lesions in cranial MRI and in the tests performed for the differential diagnosis; these patients were diagnosed as having brucellosis and were considered to have small vessel vasculitis. One patient was admitted with clinical signs of meningoencephalitis and abscess. Although cranial nerve involvement is expected in NB, vestibulocochlear nerve involvement is most commonly seen in 10% (Gul et al., 2009). Bilateral hearing loss was present as cranial nerve involvement in four of the nine patients. Audiometric examinations were not performed in the other patients because they did not describe hearing loss. One patient had two‐sided horizontal view limitation, and one had epileptic seizures.

Three types of anomalies have been described in cranial imaging; inflammation, white matter changes, and vascular events. Granulomatous lesions occur as a result of inflammation. White matter changes are thought to develop due to an autoimmune origin, and they appear as T2W hyperintense lesions. These lesions may be diffuse with the involvement of arcuate fibers or may be seen as focal demyelination or periventricular involvement. These lesions can mimic infectious lesions such as Lyme disease, or inflammatory such as multiple sclerosis (MS) and acute disseminated encephalomyelitis (ADEM), and finally, vascular events secondary to inflammation may cause lacunar infarcts, hemorrhagic strokes, or venous thromboses (Al‐Sous, Bohlega, Al‐Kawi, Alwatban, & McLean, 2004).

NB causing cerebral venous thrombosis has been described in the literature (Faraji, Didgar, Talaie‐Zanjani, & Mohammadbeigi, 2013). It is also known to cause arterial vasculopathy (Adaletli et al., 2006; Al‐Sous et al., 2004). It causes granulomatous vasculitis, especially in the skin veins and aorta. Cranial granulomatous lesions are very rare (Al‐Sous et al., 2004). Different mechanisms related to the cerebrovascular involvement of brucellosis have been described. One may be a hemorrhagic stroke resulting from the rupture of the mycotic aneurysm originating from brucellosis endocarditis, or an inflammatory process of the vessels or venous system resulting in lacunar infarcts, small hemorrhages, or venous thromboses (Adaletli et al., 2006; Bingöl & Togay‐Işıkay, 2006; Hansmann & Schenken, 1932; Pascual, Combarros, Polo, & Berciano, 1988). Some cases of cerebral vasculitic findings in MR angiography or digital subtraction angiography have been reported; however, patients with no findings have also been reported (Adaletli et al., 2006; Al Deeb, Yaqub, Sharif, & Phadke, 1989). In our cases, there were three cases of ischemic stroke due to large vessel involvement, but there was no hemorrhagic stroke. In two of our young patients with stroke, MR and CT angiography were compatible with vasculitis. In another patient MR angiography, a saccular aneurysm was detected. There are publications in the literature reporting that NB may rarely cause aneurysm (Kaya et al., 2008). In the other case, MR angiography or DSA was not performed. In five patients, white matter changes were present, and in the tests performed in these patients for the differential diagnosis of MS, vasculitis, and bronchial plexus granulomas, brucellosis was detected (Al‐Sous et al., 2004; Ciftci, Erden, & Akyar, 1998; San Miguel et al., 2006; Sohn et al., 2003). Cerebral granulomas were detected in two of our cases, and in one case, there was partial regression of a granuloma with treatment in the follow‐up MRI. One of our patients was followed up with a diagnosis of meningoencephalitis, and a brain abscess developed due to irregular drug use. There are rarely defined such cases in the literature (Türel et al., 2010). In all of our cases, the Rose Bengal and tube agglutination tests were positive, and positivity was detected for two of the six patients in whom we could perform LP for CSF examinations. However, there was no positivity in the CSF cultures of these patients. Blood cultures were negative in all nine cases. Consistent with the literature, our study showed that the tube agglutination test was more sensitive than the Rose Bengal test, and blood or CSF culture positivity was high but sensitivity was low.

The normal range of CSF protein in CSF analysis is 15–45 mg/dl. In a study conducted by Karsen et al. (2012), the content of CSF protein was determined as 118.4 ± 18.3 mg/dl. In our study, the six patients on whom we performed LP had CSF protein levels of 272, 87, 618, 15.9, 30, and 243 mg/dl, respectively; the average level was 211 mg/dl. This may be because our patients were in the chronic phase of the disease.

The normal range of the CSF/serum glucose ratio is 0.50–0.66. In the study performed by Karsen et al. (2012), the CSF/serum glucose ratio was found as 0.42 ± 0.14. In our study, the ratio of CSF/serum glucose in our six patients who underwent LP was found as 0.45, in agreement with the literature.

The clinical presentation of NB is quite varied and can mimic many diseases. For this reason, blood and CSF are important for NB in the differential diagnosis of chronic infections such as tuberculosis and Lyme disease, especially in endemic areas. NB should be remembered in the differential diagnosis of demyelinating diseases such as MS, because of the existence of the immunoglobulin (Ig) ‐G content and oligoclonal band formation can be also seen in NB (Shakir, 1986).

Although there is no specific guidance in the treatment of NB, many authorities maintain the combination regimen of doxycycline and two or more antibiotics for several months, depending on the response to treatment (Buzgan et al., 2010). It is reported that doxycycline, rifampicin, and trimethoprim/sulfamethoxazole (TMP/SMX) are effective in the treatment of NB because of its good penetration into the CNS (Young, 2006). Treatment regimens including third‐generation cephalosporins with good penetration into the CNS, such as ceftriaxone, are also available for NB (Buzgan et al., 2010). Steroid use is still recommended in patients with polyradiculoneuropathy, spinal, and/or cerebellar involvement (Shakir, 1986), although its efficacy is poor and there are no adequate controlled studies. Our patients were treated with rifampicin + doxycycline, rifampicin + ciprofloxacin + TMP/SMX, and streptomycin + doxycycline + TMP/SMX combinations for at least 6 months. One patient was untreated because they were lost in the acute phase, and one developed an abscess in the pons due to inadequate treatment. In another case, widespread chronic cranial white matter changes were present due to inadequate treatment. Clinical improvement was achieved in the other treated patients, and no relapses were observed.

## 5 CONCLUSION

Brucellosis still remains an important health problem in our country. Approximately 5% of patients with brucellosis develop NB due to CNS involvement causing significant morbidity and mortality. NB mimics many neurologic diseases and should be remembered in the differential diagnosis because the mortality and morbidity of the disease can be reduced with early diagnosis and treatment. Brucellosis should be kept in mind in patients with unexplained neuropsychiatric findings in areas where brucellosis is endemic. The fight against this disease should be implemented with a multidisciplinary approach.

## AUTHOR CONTRIBUTIONS

S.A.T contributed to concept of the study; M.Y. designed the study; S.Y. and F.S. supervised the study; S.H. and F.S. collected data of the materials and processed the data; S.A.T. and M.Y participated in the literature search; S.A.T. wrote the manuscript; N.Y critically reviewed the manuscript.

## CONFLICT OF INTEREST

None to declare.
